# Normal serum levels of immune complexes in postpolio patients

**DOI:** 10.1016/j.rinim.2014.06.001

**Published:** 2014-06-18

**Authors:** Eva Melin, Azita Sohrabian, Johan Rönnelid, Kristian Borg

**Affiliations:** a Division of Rehabilitation Medicine, Department of Clinical Sciences, Karolinska Institutet, Danderyd University Hospital, Stockholm, Sweden; b Department of Immunology, Genetics and Pathology, Uppsala University, Uppsala, Sweden

**Keywords:** Postpolio syndrome, Immune complexes, C1q binding immune complexes, Neuro inflammation, Autoimmunity

## Abstract

**Objective:**

The pathophysiology of the postpolio syndrome is not fully understood. Increased cytokine levels in cerebrospinal fluid and peripheral blood indicate a systemic inflammatory process. Decreased cytokine levels and the clinical effect of intravenous immunoglobulin treatment further indicate an inflammatory/immunological pathogenesis. The aim of the present study was to evaluate whether an autoimmune process follows the initial infection, by means of analyzing immune complexes.

**Patients and methods:**

Circulating immune complexes were analyzed from blood samples of 20 postpolio patients and 95 healthy controls. To compensate for differences in age between patients and controls, a sub-analysis was performed using only the 30 oldest controls. Tumor necrosis factor-inducing properties of polyethylene glycol-precipitated immune complexes were compared between the postpolio patients and 10 healthy controls.

**Results:**

When comparing levels in postpolio patients to the whole control group, including the 30 oldest investigated, there were no statistically significant differences. No difference was found in tumor necrosis factor levels induced by immune complexes when comparing patients and controls.

**Conclusions:**

There was no increase in circulating immune complex or in tumor necrosis factor-inducing effects of circulating immune complex between postpolio patients and healthy controls, indicating that the postpolio syndrome is not due to an autoimmune reaction.

## 1 Introduction

Acute poliomyelitis affects the anterior horn cells causing a flaccid paralysis. After recovery from acute polio and a stable period of at least 15 years, new or increasing symptoms including muscular atrophy, pain, weakness, and fatigue, may develop, a condition called postpolio syndrome (PPS) [1,2].

Earlier studies have shown that an on-going denervation–reinnervation process results in an increase in the motor unit size [3]. When the size of the motor unit areas reaches an upper limit and denervation is not compensated by reinnervation, there is a decrease of muscle strength. However, the pathophysiology for the on-going denervation process is not yet fully understood [2].

Various explanations have been presented including a normal ageing process, genetic predisposition and immunological factors [2,4–7]. An increase of cytokine levels in cerebrospinal fluid (CSF), and peripheral blood (PB) have been reported in PPS patients, indicating a systemic inflammatory process in PPS [5–8]. An inflammatory process is further supported by the findings of a recently published study, where PPS patients displayed a disease-specific expression of five distinct proteins [9], and in one study where PPS patients were found to have elevated leukocyte myeloperoxidase activity [10]. Furthermore, a decrease in cytokine levels and a clinical effect of intravenous immunoglobulin (IVIg) treatment further argues for an inflammatory/immunological pathogenesis of PPS [8,11–14].

One plausible explanation for the inflammatory process in PPS could be an autoimmune process initiated by the polio infection.

Immune complexes (IC) containing antibodies and their corresponding antigens are produced during normal immune responses as means of eliminating foreign substances, e.g. during infections [15]. In autoimmune diseases, such as systemic lupus erythematosus (SLE), autoantibodies associate with the corresponding autoantigens producing circulating IC [16]. Increased circulating IC levels have been found in type I diabetes [17,18] as well as in rheumatoid arthritis [19]. Thus, increased IC levels can be found in various diseases, irrespective of the presumed initial pathogenic events. Whereas SLE is regarded as the prototype IC-mediated type III disease, other diagnoses with presumed autoimmune pathogenesis, like diabetes mellitus and rheumatoid arthritis, are usually regarded as T-cell dependent diseases sorted in to the type IVa group according to the modified Coombs & Gell classification [20]. IC-associated disease can be associated with severe pain [21], and PPS patients frequently report pain [22]. The pathogenic mechanisms relating IC to pain are at present unknown. In clinical studies of IVIg treatment of PPS patients a positive clinical effect has been shown regarding pain and it has been suggested that the decrease in pain may be the primary effect of IVIg in PPS [14].

In the Swedish population, immunity against poliomyelitis virus is high. Böttiger and colleagues found that antibodies against the three types of polioviruses were present at the dilution ≥1:4 in over 95% of the studied population [23]. In the PPS population, antibodies against all three polioviruses were still significantly higher than in control groups consisting of patients with Multiple Sclerosis and other neurological diseases (K. Borg, personal communication 2014). There is, thus, a potential pathophysiological explanation for the inflammatory process in PPS, i.e. the initial poliovirus infection. There are also indications that a pathological reaction to the infection may occur. The aim of the present study was thus to evaluate whether the initial infection is followed by a delayed exacerbated IC response, which would suggest an autoimmune process. We have investigated circulating IC levels both by a conventional C1q binding technique, as well as by analyzing the cytokine-inducing properties of polyethylene glycol (PEG)-precipitated IC, as earlier performed in investigations of patients with SLE [24–27].

## 2 Patients and methods

### 2.1 Patients and controls

Circulating IC were analyzed in 20 PPS patients (17 women and 3 men, mean age 64 years (range 38–79 years)) recruited from the postpolio outpatient clinic, University Department of Rehabilitation Medicine at Danderyd University Hospital, Stockholm, Sweden. The patients fulfilled the criteria for PPS, and participated in rehabilitation groups specialized for postpolio syndrome. None of the patients were currently treated with IVIg therapy. Serum samples were stored at −20 °C until analyzed. The subjects׳ consent was obtained according to the declaration of Helsinki, and the study was approved by the Regional Ethical Review Board in Stockholm, Sweden.

As negative controls 95 healthy blood donors from the department of clinical immunology and transfusion medicine at Uppsala University Hospital were used (mean age was 42 years (range 20–69 years) 36 women, 59 men). In order to compensate for the difference in age between patients and controls, a sub-analysis was performed using only the 30 oldest controls (with a mean age of 56 years, 15 women, 15 men (range 50–69 years)). As positive controls, data on 162 SLE sera previously investigated with the same technique were used. Samples had been stored at −70 °C.

### 2.2 Measurement of circulating C1q-binding IC

Measurement was performed using the Quanta Lite C1q CIC ELISA (Innova Diagnostics, San Diego, CA), according to the manufacturer׳s instructions. The lowest point on the standard curve was 1.23 µg Eq/ml, and the analytical sensitivity was 0.1 µg Eq/ml. In an evaluation performed by the manufacturer, 9/200 (4.5%) of healthy blood donors had increased levels of CIC>10.8 µg Eq/ml.

### 2.3 Measurement of TNF induction by PEG-precipitated IC

This procedure was performed as previously described [26]. Sera were incubated at 4 °C overnight with equal volume phosphate buffer saline (PBS) pH 7.4 containing 5% PEG 6000 and 0.1 M EDTA. One mIliter PBS containing 5% human serum albumin (HSA) and 2.5% PEG was added to 1.5 ml autoclaved Eppendorf tubes. Autoclaved plastic cylinders made from 5 ml pipette tips were introduced into the Eppendorf tubes. 1:3 diluted serum in RPMI-1640 containing 2.5% PEG, were gently placed on the top of PBS–HSA–PEG layers. The tubes were centrifuged at 2100*g* and 4 °C for 20 min. Supernatants of the less dense RPMI-1640 solution and the remaining PBS–HSA–PEG were removed and the PEG-precipitates were dissolved with ice-cold sterile PBS to the initial volume of serum and stored on ice before cell stimulation. Meanwhile, buffy coats from healthy blood donors were diluted 1:4 with sterile PBS and peripheral blood mononuclear cells (PBMC) were separated using Ficoll Paque Plus density gradient (GE Healthcare Biosciences AB, Uppsala, Sweden). After removal of supernatants the PBMC were washed twice with sterile PBS. Mononuclear cells were counted and diluted to 1.1×10^6^ cells/ml in cell culture medium RPMI-1640 (Gibco, Life Technologies, Stockholm, Sweden) supplemented with 1% HEPES, 1% penicillin streptomycin, 10% fetal calf serum (FCS) and 0.5 µU polymyxin B sulfate. Then, 270 µl PBMC were added to 96-well polystyrene culture plate and freshly prepared PEG-precipitates were transferred to the wells (10% vol/vol). After 20 h of incubation in a 5% CO_2_ incubator, the supernatants were collected and the level of cytokine induction by IC were measured in a TNF-α ELISA using the anti hTNF-α mouse monoclonal IgG_1_ (MAB610) capture antibody and the biotinylated hTNF-α goat IgG (BAF210) detection antibody (R&D Systems, Minneapolis, USA). Due to technical failure, we obtained no PEG precipitates for two PPS patients as well for one SLE patient.

### 2.4 Statistics

Data from the patient and control groups were compared using the non-parametric Mann–Whitney’s *U* test. *P* values <0.05 were considered as significant.

## 3 Results

**Fig. 1:**
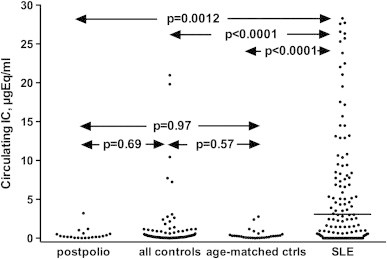
Distribution of immune complex levels among 20 PPS patients, 95 healthy controls, 30 healthy controls with the best age-match compared to the PPS patients, and 162 SLE patients.

**Fig. 2:**
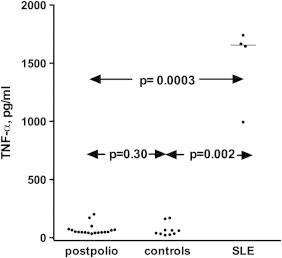
Distribution of supernatant levels of TNF induced by PEG-precipitated immune complexes from 18 PPS patients, 10 healthy controls, and 4 SLE patients with known high IC levels.

There was no difference in levels of circulating IC when values of PPS patients were compared to those of the controls, including the group with the 30 oldest controls (*p*=0.69 and *p*=0.97 respectively). Levels of IC were significantly higher among the SLE patients as compared to the levels of PPS patients and controls (*p*=0.0012 and *p*=0.0001 respectively) (Fig. 1). There was no difference in levels of circulating IC between female and male control subjects (data not shown). When the occurrence of IC was investigated by their cytokine-inducing properties no difference was found between 18 PPS patients and 10 healthy controls. Supernatant levels of TNF-α were on the other hand significantly higher when PBMC had been stimulated with PEG-precipitates from four SLE sera with known high IC levels (Fig. 2).

## 4 Discussion

The aim of the present study was to determine whether or not there might be an autoimmune reaction causing PPS. The background to the study was that data from previous studies indicate an inflammatory process in cerebrospinal fluid as well as peripheral blood in PPS patients [5,6,8,9,11]. High levels of antibodies, and a positive clinical effect when the inflammation is dampened by immunological treatment, also indicate an active inflammatory process in PPS [13], and it was speculated that the inflammation is secondary to an autoimmune process. As shown from the results in the present study there was no increase in IC in PPS patients when data of the patients were compared with those of healthy controls. IC was measured both by biochemical means, as levels of IgG-containing IC binding to C1q, and by functional means, as levels of TNF-α induced *in vitro* by PEG-precipitated IC. In both these investigations, SLE sera known to contain elevated levels of IC yielded high responses.

There is a possibility that PPS patients might have increased circulating IC containing mainly IgA or IgM. Although IC containing IgM (but not IgA) would bind to C1q, they would still not be detected by the IgG-specific secondary antibody in our ELISA. We also think that IC containing only non-IgG isotypes would show negative reactivity in our functional test, as our previous studies have shown IC to induce cytokines via the IgG-specific FcγRIIa receptor [24,26].

A weakness of the study is the different sex distribution in PPS patients and controls, but as the analysis did not show any difference in IC levels between the female and male controls, we believe that the comparison is still valid.

Thus, further immunological studies are needed in order to increase the knowledge of the immunological pathophysiology of PPS.

## Authors׳ contributions

EM contributed to the design of the study, recruited the patients, and wrote the manuscript. AZ performed PEG precipitations, cytokine analyses and statistical calculations. JR participated in the design of the study, recruited the controls, made the ELISAs, statistical calculations and helped to draft the manuscript. KB conceived of the study, and participated in its design and coordination and helped to draft the manuscript. All authors read and approved the final manuscript.
